# Application comparison of paediatric myocardial protection procedures in arterial switch surgery

**DOI:** 10.1177/02676591241309842

**Published:** 2024-12-21

**Authors:** Frank Münch, Matthias Kohl, Nicola Kwapil, Oliver Dewald, Michela Cuomo, Ariawan Purbojo

**Affiliations:** 1 Department of Cardiac Surgery, 27168 University Hospital Erlangen, Erlangen, Germany; 2 9171 Friedrich-Alexander-University Erlangen-Nuremberg (FAU), Erlangen, Germany; 3 120263 Hochschule Furtwangen, Villingen-Schwenningen, Germany

**Keywords:** arterial switch operation, cardiopulmonary bypass (CPB), cardioplegia, microplegia, myocardial protection, paediatric

## Abstract

**Background:**

Reliable myocardial protection is essential for a good outcome after arterial switch operation.

**Patients and Methods:**

We evaluated 56 neonates with arterial switch operation in this retrospective study. Three types of cardioplegia were used: antegrade Custodiol® (CCC) *n* = 22, antegrade Custodiol® plus paediatric microplegia (mix) *n* = 14, and antegrade plus retrograde intermittently paediatric microplegia (blood) *n* = 20. We evaluated the extent of myocardial injury using troponin I, Creatine kinase (CK), CK-MB (CK in myocardial cells) and vasoactive inotrope score (VIS), immediately- and the first postoperative day, as well as outcome parameters. A statistical analysis was conducted using multiple linear regression, with adjustments made for the RACHS score and ischemia time, at a significance level of 5%.

**Results:**

Preoperative data were comparable between the three groups. Aortic cross clamp time was significantly different between the three groups (CCC: 115 ± 26 min: mix: 162 ± 35 min: blood: 153 ± 31 min). We found significantly lower troponin I release in the blood group 14 ng/mL [CI95 10; 18] versus CCC group 36 ng/mL [CI95 27; 48] and versus mix group 27 ng/mL [CI95 19; 38]; troponin I 24 h blood group 8 ng/mL [CI95 6; 11] versus CCC group 14 ng/mL [CI95 10; 19]. No significant differences were found in CK, CK-MB, VIS, as well as in outcome parameters 30-day mortality, ventilation time, hospital stay or ECMO implantation.

**Conclusions:**

Intermittent paediatric microplegia led to a significantly lower release of troponin I, despite significantly longer ischemia times than after application of Custodiol®. Paediatric microplegia can be safely performed in neonates and also offers the advantage of miniaturization of the Cardiopulmonary bypass setup.

## Introduction

Neonates with transposition of the great arteries (TGA) should ideally be corrected in the first week of life by arterial switch operation (ASO) on cardiopulmonary bypass.^1^ The anatomical correction of this congenital heart disease was first performed by Jatene in 1975.^2^ A meta-analysis showed a >20 years survival rate of 89% (CI95: 80–95%) with only 692 patients included. A combined analysis of TGA patients treated with ASO showed a pooled medium-term survival rate (1–20 years) of 93% (CI95: 91–94%) in 8283 patients.^3^

The success of the procedure depends on the type and administration route of cardioplegia and the optimal surgical outcome. For optimal protection of the myocardium, it is necessary that the ideal cardioplegia solution induces cardiac arrest quickly and is reversible. With a low haemodilution and little systemic effect, long administration intervals and simple application. To prevent overstretching of the heart, the patient is drained at the Cardiopulmonary Bypass (CPB) and the surgeon also performs a venting procedure via the left atrium.^4^ Many surgical teams prefer a single-shot cardioplegia to avoid excessive manipulation of the tiny coronary arteries with intermittent cardioplegia application. After successful corrective surgery, reperfusion injury can occur, in particular due to the re-influx of oxygenated blood into the ischaemic myocardium. Mitochondrial calcium overload during reperfusion occurs in the subendocardial myocardium. The processes that take place lead to reduced micro perfusion and lipid peroxidation and thus to direct damage to the cell membranes and mitochondria.^4^

Despite numerous TGA studies, the most effective protocol for myocardial protection during ASO still remains controversial. In this retrospective study we compared three types and methods of cardioplegia delivery for optimal myocardial protection. The idea behind the three protection applications was to examine the individual effect and the combination of the procedures in myocardial protection in ASO.

## Methods

The local ethics committee approved the study protocol and patient approval was waived (application no. 22-36-Br). Sixty-five neonates with a diagnosis of transposition of the great arteries underwent ASO between 01/2017 and 07/2022. Patients diagnosed with hypoplastic aortic arch (*n* = 3) and double outlet right ventricle (DORV, *n* = 6) were excluded, leaving 56 neonates for analysis. Three cardioplegia methods were used for myocardial protection: antegrade Custodiol® (Köhler Chemie GmbH, Bensheim, Germany) in 22 patients (CCC group), antegrade Custodiol® plus paediatric microplegia in 14 patients (mix group) and antegrade plus retrograde paediatric microplegia in 20 patients (blood group).

**Figure 1.**
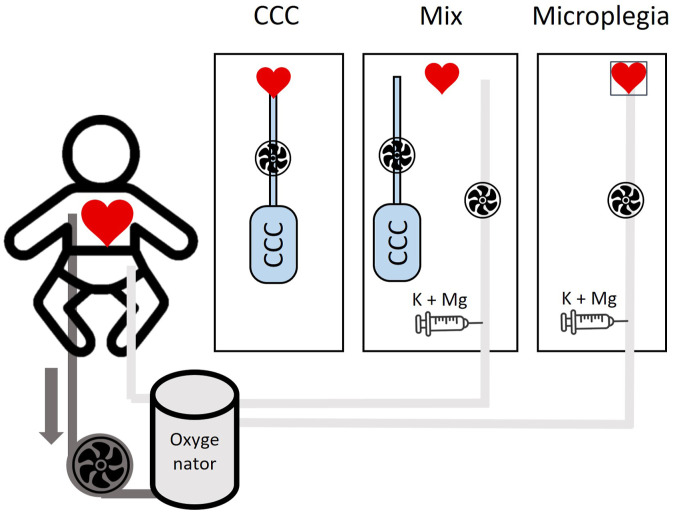
Schematic diagram of the treatment pathways.

The paediatric microplegia consisted of oxygenated blood and the addition of potassium (K) 20 mL (2 mmol/mL potassium chloride 14.9%; B. Braun Melsungen, Germany) and magnesium (Mg) 10 mL (4 mmol/mL Mg Verla® i.v. 50%; Verla Pharm, Tutzing, Germany) from a syringe pump. The ratio of blood to “cardioplegia solution” is initially 63:1 for 2 min (25 mmol/L K with 11 mmol/L Mg), first repeat-dose 83:1 (20 mmol/L K; 8.5 mmol/L Mg) and possibly a further maintenance-dose every 20 min of 120:1 (15 mmol/L K; 6 mmol/L Mg).^5^ Of these 56 neonates, 11 presented with a ventricular septal defect (VSD) which was closed during the surgery (CCC: *n* = 1; mix: *n* = 4; blood: *n* = 6) (Figure 1).

The application temperatures for cardioplegia in the present study were 4°C for Custodiol®, directly from the refrigerator. For paediatric microplegia, the blood temperature from the arterial line of the CPB was always used, which was on average 25°C above all groups.

Additional cooling of the myocardium with ice and cold water for regional myocardial cooling wasn’t used.

The antegrade line pressure, measured before the administration line behind the cardioplegia pump, after Standard Operating Procedure (SOP) was 50–60 mmHg with sufficient aortic valve closure. The retrograde application was administered at a line pressure of 35–45 mmHg.^6^

The administration of the antegrade Custodiol® was always performed antegrade via the aortic root for 5 min. After connecting or switching on the coronaries, a repeat dose antegrade of 15 mL/kg Bodyweight CCC was administered to patch the pulmonary artery and close any intracardiac defect. For the mix group, antegrade Custodiol® was initially administered via the aortic root for 5 min. After switching the coronary arteries, the first cardioplegia repeat dose was administered as antegrade paediatric microplegia, also via the aortic root for 2 min. These microplegia doses were given every 20 min until all intracardiac defects had been corrected or until the pulmonary artery had been reconstructed. The aortic clamp was then opened to release perfusion to the heart. In the blood cardioplegia group, initial antegrade paediatric microplegia was applied to the aortic root for 2 min. Then by opening the atrium and placing a purse-string suture around the coronary sinus to fix the retrograde cardioplegia catheter. An arteriotomy cannula no. 31103 (3 mm) or no. 31102, (2 mm) was used as the retrograde catheter (Medtronic GmbH, Meerbusch, Germany). Until switching on the coronaries, retrograde microplegia was administered every 20 min according to the described schema. Subsequently, myocardial protection was repeated antegrade every 20 min until surgical correction was finalised.

Cardioplegia application volume over ischemia time was documented to assess haemodilution. For qualitative assessment of myocardial protection troponin I (Trop I), creatine kinase (CK) and cardiac/myocardium creatine kinase isoenzyme (CK-MB) and Vasoactive Inotrope Score (VIS) were compared immediately postoperatively and on the first postoperative day (Trop24 h, CK24 h, CK-MB24 h, VIS24 h). We evaluated outcome parameters ventilation time, hospital stay, ExtraCorporal Membrane Oxygenation (ECMO) and 30-day mortality.

We evaluated the extent of myocardial injury using troponin I, CK and CK-MB, and Vasoactive Inotrope Score at two postoperative time points.

### Anaesthesia

After pre-operative sedation with midazolam and esketamine, anaesthesia was induced with fentanyl and propofol. After muscle relaxation with vecuronium, nasal-tracheal intubation was performed and mechanical ventilation was started. A central venous line was then inserted into the superior vena cava through the percutaneous puncture of the jugular vein. An arterial cannula was inserted into the right brachial artery to monitor arterial blood pressure.

### Cardiopulmonary bypass

CPB was performed on a modified neo heart-lung machine based on the S5 (LivaNova, München, Germany) and using the D100 complete set (LivaNova, Mirandola, Italy). The CPB set was primed and deaerated in a standardized manner with 500 mL Jonosteril (Fresenius Kabi, Bad Homburg, Germany) and 5000 I.U. heparin (ratiopharm GmbH, Ulm, Germany). The excess full electrolyte mixture was drained into the waste bag and then the priming solution was supplemented with 5 mL sodium bicarbonate 8.4% (Serag Wiessner, Naila, Germany), and 250 mg vitamin C (Centricor^®^, Wörwag Pharma GmbH, Germany) as a free radical scavenger. The total priming volume was 147 mL. A part of the priming solution was replaced by packed red blood cells (PRBC) in cases with calculated haemoglobin value (Hb) below the transfusion trigger threshold of 8.5 g/dl after the start of the CPB. The body surface area (BSA) of the patient was calculated according to DuBois formula. The estimate blood flow target was 3 L/min/m^2^BSA. Nitroprusside sodium was administered as an intraoperative volume pooling at a rate of 0.5–1.5 µg/kg/min from the beginning of CPB until at least the end of modified ultrafiltration (MUF).^7^ NIRS (Near-infrared spectroscopy) sensors were used to monitor perfusion on the left and right side of the forehead and on the right kidney during CPB.^8^

### Surgical procedure

The surgical procedure was performed through standard median sternotomy and cardiopulmonary bypass (aortic and bi-caval cannulation). The mean body temperature was approximately 25°C. The first cardioplegia application was administered into the aortic root, and subsequent doses varied between ante- and retrograde application in the three groups according to the study design. To avoid tension following the Lecompte manoeuvre, both pulmonary arteries were mobilized into the pulmonary hilus. The pulmonary artery was divided before the pulmonary bifurcation, and the ascending aorta was divided distal to the sinotubular junction. Next, we performed the Lecompte manoeuvre. The coronary artery buttons were then excised from the sinuses, maintaining enough cuff aortic tissue. After marking the commissure, the neo aorta was reconstructed through end-to-end anastomosis. Two new coronary ostia were created in the neo aortic root, avoiding commissures, and the coronary bottoms were re-implanted. The atrial septal defect was closed either directly or with an autologous pericardial patch. If a ventricular septal defect was present, it was approached through the tricuspid valve and closed with a patch. Following the reconstruction of the main pulmonary artery with an autologous pericardial patch, the pulmonary trunk was reconnected to the pulmonary bifurcation (end-to-end anastomosis) on beating heart.

### Catecholamine requirement

For better comparability of vasoactive support, the catecholamine requirement was determined using the Vasoactive Inotrope Score.^9^

VIS=(100*([value Perf. Epi]*0.1))+(100*([value Perf. Nor]*0.1))+(10*([value Perf. Milrinon]*0.75))

### Data collection and statistics

The statistical analysis was performed using R 4.3.3.^10^ Since there was no a priori sample size calculation, we instead determined the effect sizes in form of Cohen’s^11^ d that can be detected in the present study for the given sample sizes, at a power of 80% and a significance level of 5%. The effect size of d: 0.2 – 0.5 corresponds to a small effect, d: 0.5 – 0.8 would be a medium effect and >0.8 would be a strong effect. The demographic and intraoperative data was analysed by 1-way ANOVA approaches (Welch 1-way ANOVA, Kruskal-Wallis Test) or Fisher’s exact test followed by corresponding post-hoc tests (pairwise Welch t-, Wilcoxon-Mann-Whitney or Fisher’s exact tests) if a significant difference was detected. The laboratory parameters were analysed by multiple linear regression, adjusting for RACHS-score and ischemia-time followed by post-hoc tests using R package emmeans^12^ if significant differences between the myocardial protection procedures were detected. The laboratory parameters Troponin, CK and CK-MB at 0 h and 24 h as well as the VIS-score at 0 h, ventilation-time and intensive-care duration were log10-transformed, which stabilized the variances and normalized the distributions. The need for ECMO and 30-day mortality were analysed using multiple logistic regression. In all cases, diagnostic plots were used to verify distributional (qq-plots) and other model assumptions (residual plots) by using R packages qqplotr^13^ and performance.^14^ All tests were two-sided and the significance level was set at 5%. The *p*-values of the post-hoc tests were adjusted for multiple testing by the method of Holm.^15^ 95% confidence intervals (CI95) were not adjusted.

## Results

The effect sizes that can be detected for the given sample sizes at a power of 80% and a significance level of 5% are large for all three comparison groups (CCC vs mix: d = 0.99; CCC vs blood: d = 0.89; mix vs blood: d = 1.01). The detection of medium or even small effect sizes is therefore unlikely in the present study. The preoperative data were comparable between the groups. However, there were significant differences in the ischemia time between the CCC group (115 ± 26 min) and the mix group (162 ± 34 min) (*p* >.001; CI95 −72; −22), as well as between the CCC group (115 ± 26 min) and the blood group (153 ± 31 min) (*p* >.001; CI95 −61; −16). Additionally, the volume of cardioplegia was significantly lower in the blood group (8 ± 2 mL) compared to the CCC group (340 ± 80 mL) and mix group (293 ± 60 mL) (Table 1).

**Table 1.** Demographics and intraoperative data 1-way ANOVA approaches (Welch 1-way ANOVA, Kruskal-Wallis Test) or Fisher’s exact test with post-hoc; significant pairs marked as (ns, *, *) are for the comparisons CCC versus mix, CCC versus blood and mix versus blood (significant codes: 0 ‘***’ 0.001 ‘**’ 0.01 ‘*’ 0.05 and ns > 0.05).

Demographic data		CCC		Mix		Blood		Sig.
		*n* = 22 (+VSD: *n* = 1)		*n* = 14 (+VSD: *n* = 4)		*n* = 20 (+VSD: *n* = 6)		
		Mean	CI95	Mean	CI95	Mean	CI95	*p*
Age	(d)	7.3	5.9; 8.8	6.2	5.0; 7.4	8.0	5.4; 10.5	.280
Weight	(g)	3477	3249; 3705	3476	3220; 3732	3084	2816; 3351	.048 (ns, ns, ns)
Gender	(m/w)	(16/6)		(10/4)		(12/8)		.716
RACHS		3.0	3.0; 3.0	3.0	3.0; 4.0	3.0	3.0; 4.0	.127
Intraoperative								
Cardiopulmonary bypass	(min)	201	179; 223	254	221; 286	244	217; 271	.010 (*, *, ns)
Ischemia-time	(min)	115	103; 126	162	142; 182	153	139; 168	<.001 (***, ***, ns)
Lowest temp.	(°C)	24.9	22.3; 27.5	24.3	22.9; 25.7	25.5	24.2; 26.8	.444
Haemofitration	(Yes/no)	(14/6)		(14/0)		(19/1)		.021 (ns, ns, ns)
Modified ultrafiltration	(Yes/no)	(18/2)		(14/0)		(20/0)		.329
Cardioplegia	(mL)	340	305; 376	293	258; 327	8	7; 9	<.001 (*, ***, ***)

**Figure 2.**
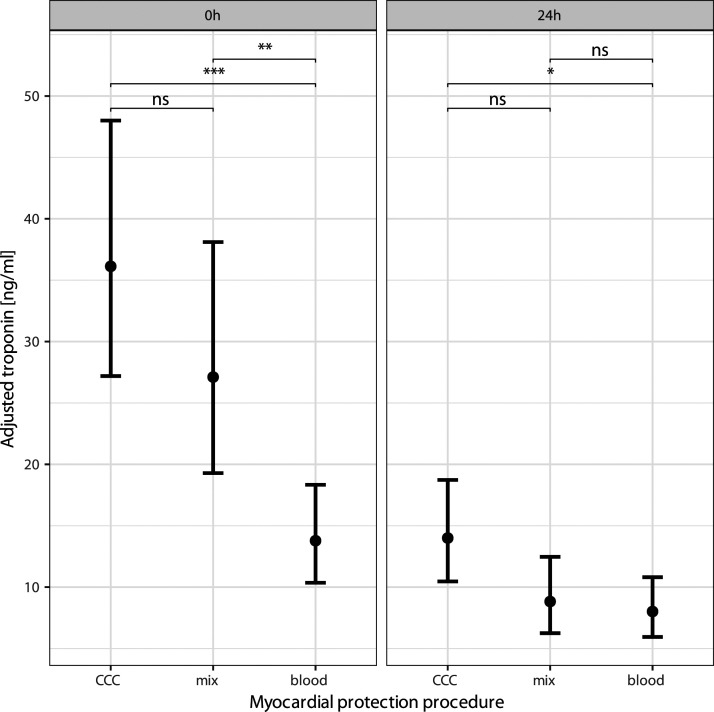
Troponin values are adjusted for RACHS-score and ischemia-time using the marginal means approach of R package emmeans (Signif. codes: 0 ‘***’ 0.001 ‘**’ 0.01 ‘*’ 0.05 and ns >0.05).

Analysis revealed significant differences in the postoperative cardiac marker troponin I in the CCC group versus blood group: (36 ng/mL [CI95 27; 48] vs 14 ng/mL [CI95 10; 18], *p* < .0001), and versus mix group: (27 ng/mL [CI95 19; 38], *p* = .2); blood group versus mix group: (14 ng/mL [CI95 10; 18] vs 27 ng/mL [CI95 19; 38], *p* = .0001). Trop24 h in the CCC group versus blood group: (14 ng/mL [CI95 10; 19] vs 8 ng/mL [CI95 6; 11], *p* = .03), and versus mix group: (9 ng/mL [CI95 6; 13], *p* = .1); blood group versus mix group: (8 ng/mL [CI95 6; 11] vs 9 ng/mL [CI95 6; 13], *p* = .6) (Figure 2). The parameters CK, CK-MB and VIS (catecholamine requirement) did not show significant differences at any time points (Table 2).

**Table 2.** Laboratory parameters (multiple linear regression); VIS = vasoactive inotrope score and VIS24 h = VIS after 24 h.

Laboratory data		CCC		Mix		Blood		Sig.
		*n* = 22 (+ VSD: *n* = 1)		*n* = 14 (+ VSD: *n* = 4)		*n* = 20 (+ VSD: *n* = 6)		
		Mean	CI95	Mean	CI95	Mean	CI95	*p*
VIS		9.0	7.4; 10.9	10.7	8.6; 13.4	8.1	6.3; 10.4	.174
CK	(I.U.)	2000	1611; 2483	1690	1232; 2317	1462	1206; 1773	.171
CK-MB	(I.U.)	223	186; 267	177	135; 231	179	146; 221	.540
VIS24 h		5.9	4.2; 7.6	7.8	3.6; 12.1	7.7	6.1; 9.4	.988
CK24 h	(I.U.)	963	765; 1214	975	739; 1285	852	650; 1117	.294
CK-MB24 h	(I.U.)	91	72; 114	81	61; 106	80	61; 104	.372

The 30-day mortality (CCC: *n* = 1) and other outcome parameters were also not significantly different (Table 3).

**Table 3.** Patient outcome (multiple logistic regression).

Outcome		CCC		Mix		Blood		ANOVA
		*n* = 22 (+VSD: *n* = 1)		*n* = 14 (+VSD: *n* = 4)		*n* = 20 (+VSD: *n* = 6)		
		Mean	CI95	Mean	CI95	Mean	CI95	*p*
ventilation-time	(h)	38	29; 51	54	31; 94	44	30; 64	.613
Intensiv-care	(d)	10	8; 14	14	11; 17	11	7; 17	.529
ECMO	(Yes/no)	(1/21)		(1/13)		(2/18)		.641
30 d mortality	(Yes/no)	(1/21)		(0/14)		(0/20)		.201

Regardless of the cardioplegia employed, a significant correlation was observed between the length of the ischemic-time and the catecholamine requirement (VIS24 h, *p* = .04), the duration of ventilation-time (*p* = .006), and the length of the intensive-care stay (*p* = .006).

## Discussion

In this present study employed the new paediatric microplegia,^5^ which provided better myocardial protection despite a significantly longer ischemia time (mean approximately 40 min). The increased incidence of more complex TGAs with VSDs in the microplegia group, which were also treated during correction, resulted in significantly longer ischemia times in this group. Troponin level were significantly lower immediately postoperatively and absent on the first post-operative day. As troponin is considered the gold-standard for assessing myocardial damage,^16,17^ this study demonstrates that intermittent ante- and retrograde microplegia application can improve myocardial protection. The combination of Custodiol® and microplegia resulted in higher postoperative troponin levels in the 14 patients compared to the plain Custodiol® or paediatric microplegia administration.

In a similar retrospective study to the one presented here, Bojan et al. analysed 218 ASO patients. They achieved myocardial protection using Custodiol® (*n* = 30) or warm microplegia (*n* = 188), which had a significantly different application and composition compared to the microplegia used in our study. Bojan et al. used a mixture of 60 parts oxygenated blood and one part potassium-magnesium-procaine (K 0.8 mmol/mL, Mg 0.8 mmol/mL, NaCl 2.45 mmol/mL and procaine 0.05 mmol/mL), which was initially applied via the ascending aorta. Subsequent doses were administered directly into the coronary ostia for 1 min every 10–12 min. Even though the two microplegia procedures in the present study and that of Bojan et al. differ in the electrolyte concentrations, both procedures are ‘microplegia’ that use blood as a carrier. The working group under Bojan postulated that the application of Custodiol®-cardioplegia in new-borns undergoing ASO is associated with a significantly higher release of troponin, which, in the opinion of the authors, indicates poor myocardial protection.^18^

In general, the use of blood cardioplegia based on microplegia or mini-cardioplegia offers several advantages. These include reduced intraoperative hemodilution, restoration of spontaneous cardiac rhythm after clamp release, lower postoperative CK-MB levels and catecholamine requirements, and shorter ICU stay. These findings were demonstrated in the meta-analysis by Owen et al.^19^

The study demonstrates the safe implementation of microplegia adapted to the myocardium in new-borns. Due to the novelty of this paediatric microplegia, a reliable long-term result in this cohort are not yet available. Our study shows improved myocardial protection and reduced haemodilution, which is particularly important in infant and child perfusion. The use of paediatric microplegia in the present study has significantly less crystalloid cardioplegia solution on average (8 mL compared to 340 mL) with significantly longer ischemia times, suggesting less haemodilution at the CPB. These findings suggest that paediatric microplegia may be a more effective option for cardioplegia-associated haemodilution in paediatric patients. Although most of the Custodiol^®^ solution is aspirated in the coronary sinus, some of it also enters the CPB, along with some erythrocytes that are lost in the aspirator.

Crystalloid-based solutions, such as Custodiol® or Del Nido, are known to cause significant haemodilution. Even the highly successful Buckberg blood cardioplegia has a 20% crystalloid content and requires a more complex CPB application setup^20^ compared to the paediatric microplegia presented in our study.^4,5^ Additionally, the use of Custodiol^®^ may lead to perioperative cardioplegia-associated hyponatremia, which should not be underestimated. To counteract this risk, we consistently aspirated the Custodiol^®^ cardioplegia from the coronary venous sinus in our study. Hyponatremia is the most frequent electrolyte abnormality in hospitalized patients and can result in cerebral oedema.^21,22^ Therefore, when using Custodiol®, it is essential to consistently aspirate the cardioplegia solution from the coronary sinus. Bojan et al conducted a study where they omitted the aspiration of Custodiol^®^ resulting in significantly higher haemodilution, haemofiltration, and hyponatremia (median to 135 mmol/L compared to 129 mmol/L) in the crystalloid group.^18^

### New perspectives for minimalized CPB-systems with microplegia

The mismatch between CPB-system and patient blood volume is particularly high in infant CPB. To minimize the degree of haemodilution, component selection and tubing length reduction on a modified CPB-set should be individually adapted to the body weight.^23–25^

Pouard et al., performed ASO on normothermic CPB with normothermic microplegia using a modified CPB-circuit without heat-exchanger, resulting in significantly lower ventilation time and troponin I compared to hypothermic CPB with cold cardioplegia. The authors suggested the lack of hypothermia as the main reason for their beneficial findings in microplegia group.^26^

This can be achieved be simplifying or minimization of CPB-system, eliminating components such as haemofilter, cardioplegia heat exchanger, etc., which can reduce contact activation, haemodilution and foreign blood administration. To prevent adverse events associated with CPB, it is recommended to implement measures that reduce interaction between the patient and the CPB system.^27^ Murin et al. reported a study on patients up to 7 kg across all heart defects on CPB without blood priming resulting in a significantly reduced mortality.^28^ Böttcher et al. described this approach as a basic requirement for “alien-blood-free paediatric cardiac surgery with CPB.”^29^ Karkouti et al. described in a meta-analysis a positive correlation between the amount of blood products transfused and the acute renal insufficiency (AKI).^30^ Sufficient haemostasis was often achieved despite minimizing of the CPB-circuit and enabling blood-free priming of the CPB-system.^23^

## Summary

The study demonstrates that optimal myocardial protection can be safely achieved even in complex procedures with long aortic cross-clamp times when using blood microplegia. In our experience, this type of myocardial protection is beneficial for several reasons. In addition, after opening the aortic clamp, the infant’s heart starts immediately without rhythm problems and has a good pumping function. To avoid additional manipulation of the tiny coronary arteries during coronary transfer, retrograde application of cardioplegia can be used instead of direct administration into the coronary arteries.

## Limitations

The investigation is a retrospective analysis of a single centre, with a small number of patients. The definition of ‘24-h’ measurement was between 9 and 10 a.m. on the first post-operative day. This defined time was chosen because of the routine laboratory collection, and is on average ‘only’ 20–21 h after admission to the intensive care unit.

## Conclusion

The present study describes that intermittent paediatric microplegia resulted in significantly less myocardial damage when compared to Custodiol^®^, and despite significantly longer ischemia times. This suggests that the new paediatric myocardial protection procedure may have beneficial effects in complex congenital disease when compared to the standard Custodiol^®^, or a combined cardioplegia procedure. Also, it may be safe to consider retrograde intermittent microplegia administration in new-borns. These results should be confirmed by other groups and in a multicentre effort in provide better evidence for optimal myocardial-protection. Future studies with microplegia in switch operations should be prospective randomised and the points of investigation should be prolonged intervals for microplegia, at 30 min and 40 min.
